# Morphology of the Tympano‐Periotic Complex in Stranded Odontocetes in Northeast Brazil

**DOI:** 10.1002/jmor.70089

**Published:** 2025-09-26

**Authors:** Gabriela Colombini‐Corrêa, Maria Morell, Ana Bernadete Lima Fragoso, Daniel Solon Dias de Farias, Flávio José de Lima Silva, Simone Almeida Gavilan

**Affiliations:** ^1^ Laboratório de Monitoramento de Biota Marinha Projeto Cetáceos da Costa Branca ‐ Universidade do Estado do Rio Grande do Norte (PCCB – UERN) Mossoró Brazil; ^2^ Centro de Estudos e Monitoramento Ambiental – CEMAM Areia Branca Brazil; ^3^ Programa de Pós‐Graduação em Biologia Estrutural e Funcional (BIOEF) Universidade Federal do Rio Grande do Norte (UFRN) Natal Brazil; ^4^ Laboratório de Morfofisiologia de Vertebrados (LABMORVE), Departamento de Morfologia, Centro de Biociências Universidade Federal do Rio Grande do Norte (UFRN) Natal Brazil; ^5^ Foundation, Institute for Terrestrial and Aquatic Wildlife Research (ITAW) University of Veterinary Medicine Hannover Hannover Germany

**Keywords:** cetaceans, ear, morphometry, stranding, taxonomy, tympanic bulla

## Abstract

Morphological descriptions of the tympano‐periotic complex (TPC) are fundamental for understanding odontocete auditory adaptations, as well as their relationships with habitat, behavior, and evolutionary processes. This study analyzed the TPC morphology of six Delphinidae species stranded along the northeastern coast of Brazil: *Peponocephala electra* (*n* = 4), *Pseudorca crassidens* (*n* = 2), *Sotalia guianensis* (*n* = 39), *Stenella attenuata* (*n* = 4), *Stenella longirostris* (*n* = 4), and *Tursiops truncatus* (*n* = 4). A total of 57 TPCs were examined, with 24 morphometric measurements taken, including two novel parameters introduced in this study. The results revealed similarities in TPC morphology among species, particularly among *S. guianensis, S. attenuata*, *and S. longirostris*, which exhibited more comparable anatomical features in the structures analyzed. Species identification was supported by distinct features: in the tympanic bone, the posterior process, inner and outer prominences, and sigmoid process; and in the periotic bone, the cochlear portion, apertures for the cochlear and vestibular aqueducts, and the transverse crest. A previously undescribed anatomical structure, termed the “mesocochlear opening,” was identified in *S. attenuata*. No remarkable ontogenetic variations were observed in the TPC of *S. guianensis*, *P. electra*, or *S. longirostris*, supporting the hypothesis that auditory structures reach full development early in life. These findings highlight key morphological features of the tympano‐periotic complex that contribute to species differentiation while providing new insights into the evolutionary and ecological adaptations of odontocetes. Furthermore, this study underscores the value of detailed morphological analyses for elucidating structural taxonomic variation and supporting future studies on the auditory capabilities of odontocetes.

## Graphical abstract

This study presents the morphological and morphometric characterization of the tympano‐periotic complex (TPC) in six odontocete species stranded along the northeastern coast of Brazil. A total of 57 TPCs were analyzed, revealing interspecific differences in key anatomical features and supporting taxonomic differentiation. A novel structure, the *mesocochlear opening*, was described in *Stenella attenuata*. These findings contribute to understanding odontocete auditory morphology and interspecies variation.

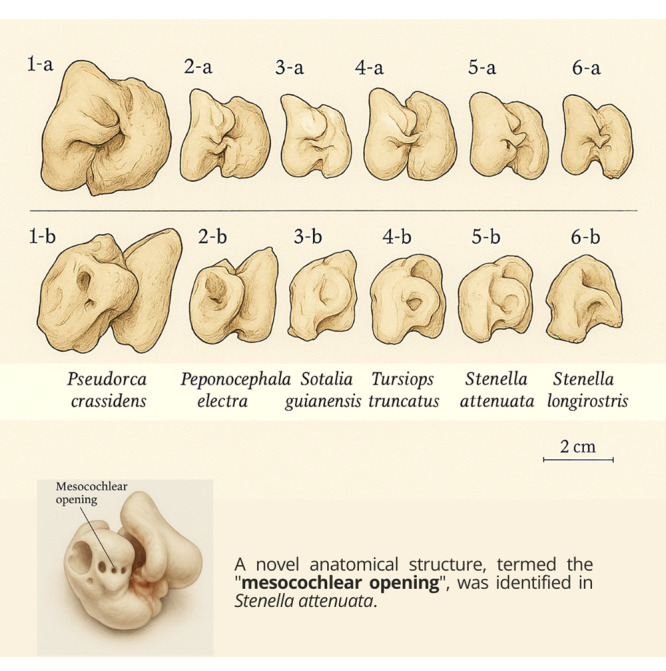

## 1 Introduction

With the transition to aquatic life, sound assumed a fundamental role in the survival of mammals. In the case of cetaceans, hearing plays a crucial role not only in communication but also in echolocation, which is essential for prey location and navigation in species inhabiting deep or highly turbid waters (Norris 1968; Tyack and Clark 2000; Ary et al. 2016). Thus, cetaceans exhibit highly complex structures for sound production and reception (Ketten 1992; Ramos et al. 2010).

The vocalizations of odontocetes exhibit a wide frequency range, reflecting their specialized adaptations for communication and echolocation. Echolocation clicks, primarily used for navigation and prey detection, typically range between 10 and 150 kHz, with some species reaching up to 200 kHz (Nummela 2009).

Whistles, which play a crucial role in social interactions, usually fall within the 1 to 30 kHz range, though some can reach up to 50 kHz (Frankel 2009). Additionally, pulsed calls, often used for group coordination and interaction, span a broad frequency range, generally between 1 and 100 kHz (Frankel 2009). This high‐frequency specialization allows odontocetes to efficiently perceive their surroundings in aquatic environments where visibility is often limited, highlighting the importance of acoustic communication in their ecological success.

The mammalian auditory system consists of three key regions: the external ear, which captures and channels sound waves; the middle ear, which transmits and amplifies these vibrations through the ossicular chain; and the inner ear (cochlea), where mechanical energy is transduced into neural impulses via sensory hair cells and associated innervation (Ketten 1992; Thewissen 2002). In cetaceans, the external ear is characterized by a small opening with a cartilaginous canal, traditionally believed to have a rudimentary function. However, recent studies have revealed the existence of mechanoreceptor cells, potentially associated with detecting pressure variations in the environment around the external canal (De Vreese et al. 2020). Sound reception in odontocetes primarily can occur via specialized perimandibular fat bodies, which are in direct contact with both the tympanic and periotic bones (Cranford et al. 2010). The incident sound waves are thought to be transmitted by these fatty tissues, which act as preferential pathways to the ear complex, especially around the ventral and/or lateral aspects of the mandible (Cranford et al. 2008; Ketten 2000; Norris 1968). The middle ear ossicles (malleus, incus, stapes) remain functionally connected, with the malleus fused to the tympanic bone, forming a continuous vibratory pathway to the inner ear, which is housed in the periotic bone. Both bones are hypermineralized and morphologically distinct, with the tympanic bone acting as a primary sound‐receiving structure and the periotic bone encapsulating the cochlea (Berta et al. 2005; Cranford et al. 2010; Ketten 2000). While the periotic bone is robust and houses the vestibular and cochlear portions, the tympanic bone resembles a U‐shaped shell in cross‐section. These two bones are interconnected by the malleus, incus and stapes ossicles, forming the tympano‐periotic complex (TPC) or tympanic bulla, highly specialized in both morphology and function (Cozzi et al. 2016).

Morphological studies of the TPC in cetaceans are still limited, reinforcing the need for further investigation to better understand its structure and functional relevance(Kasuya 1973; Ketten 1990; Parente et al. 1999; Simões‐Lopes 2006; Morell et al. 2007; Gutstein et al. 2014; Arcoverde et al. 2014; Rigon 2015; Racicot et al. 2019) and population assessments. Additionally, these studies assist in identifying anatomical and pathological variations within the complexes. Considering that the auditory sensitivity of odontocetes is directly linked to the morphology of their auditory apparatus, which may vary among different species, our study aimed to describe the morphological characteristics of the TPC in six species of odontocetes from Delphinidae that were only partially described to date. Furthermore, we sought to investigate potential ontogenetic variations.

## 2 Materials and Methods

The complexes analyzed in this study were collected from stranded deceased animals, documented by the Cetaceans of Costa Branca Project at the State University of Rio Grande do Norte (PCCB‐UERN) and the Center for Environmental Studies and Monitoring (CEMAM). The collection of cadavers was based on community reports or beach monitoring conducted as an environmental requirement for seismic prospections and oil and gas extraction activities along the coast of Rio Grande do Norte, northeastern Brazil, covering a total beach area of 410 km, between the beaches of the municipalities of São Bento do Norte (5°03′5.77″ S; 36°02′16.34″ W) and Rio do Fogo (5°16′40.81″ S; 35°22′33.16″ W).

All individuals were photographed and recorded in the PCCB/UERN database, from which, for this study, species, biometrics, age class, and sex were considered. The removal of TPC was performed during necropsy procedures, following the protocol described by Ketten et al. (2007). Subsequently, they were subjected to maceration and incorporated into the osteological collection at the Marine Biota Laboratory of the State University of Rio Grande do Norte (UERN), in Mossoró. Each complex was photographed and measured using a precision digital caliper (0.01 mm accuracy), with all measurements conducted by a single individual to minimize potential variations.

**Figure 1:**
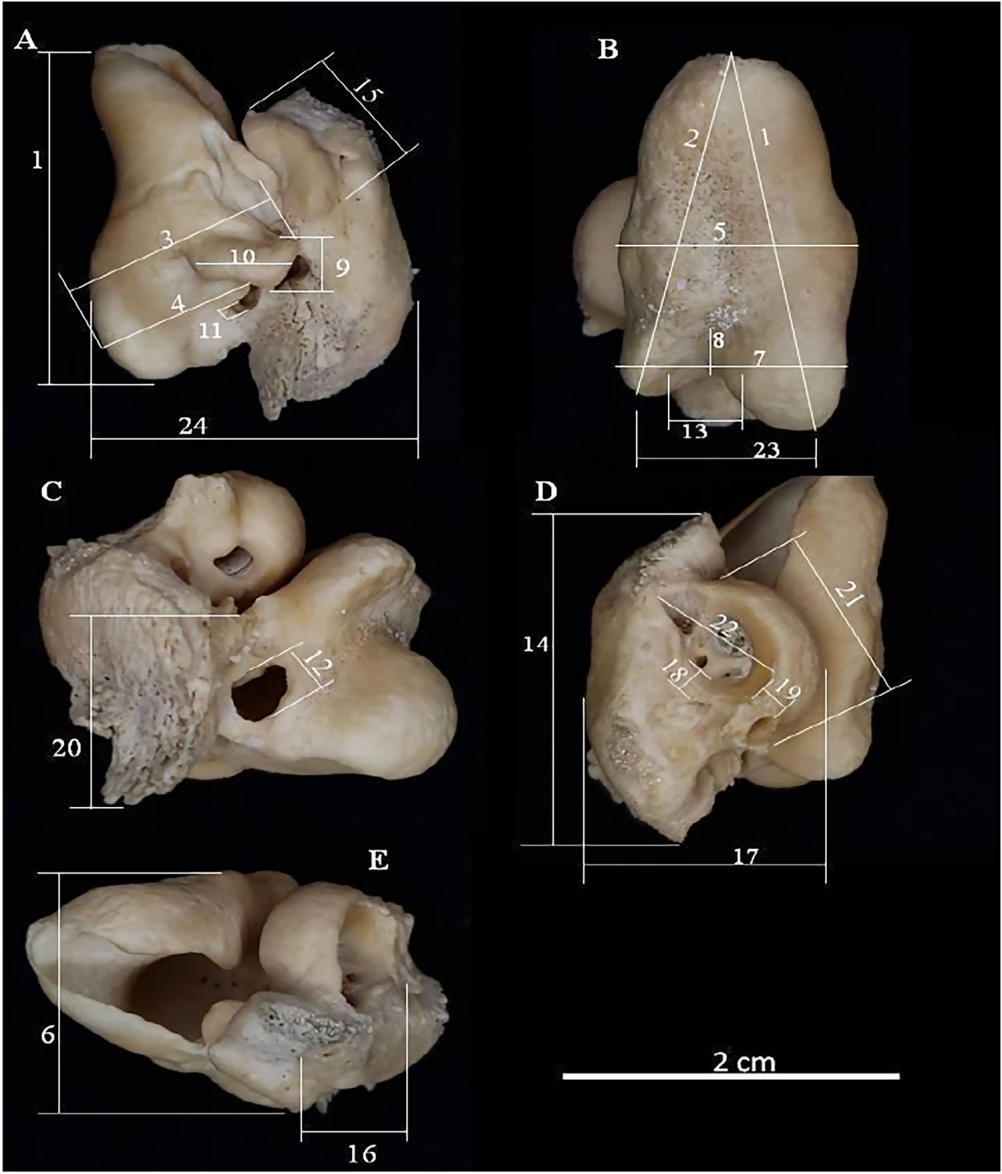
Tympano‐periotic complex of the species *Sotalia guianensis*, indicating the measurements performed: (A) lateral; (B) ventral; (C) posterior; (D) dorsomedial; (E) anterior view.

Fifty‐seven TPC from 31 individuals distributed across six species were analyzed: *Peponocephala electra* (Gray 1846), *n* = 4; *Pseudorca crassidens* (Owen 1846), *n* = 2, *Sotalia guianensis* (Van Bénéden 1864), *n* = 39; *Stenella attenuata* (Gray 1846), n = 4; *Stenella longirostris* (Gray 1828), *n* = 4; and *Tursiops truncatus* (Montagu 1821), *n* = 4. For each bulla, 24 measurements were taken (Figure 1), with numbers 1 to 22 previously described in studies by Kasuya (1973), Parente et al. (1999), and Arcoverde et al. (2014). Measurement 23 refers to the distance between the tips of the inner and outer posterior prominences of the tympanic bone, and measurement 24 refers to the width of the tympano‐periotic complex below the sigmoid process. Both were added as they lacked reference data in previous studies and seemed to exhibit potential variations among species.

Associations between the length of the animal and the maximum length of the tympanic and periotic bones were also evaluated, represented respectively by measurements M1 and M14 (Figure 1).

**Figure 2:**
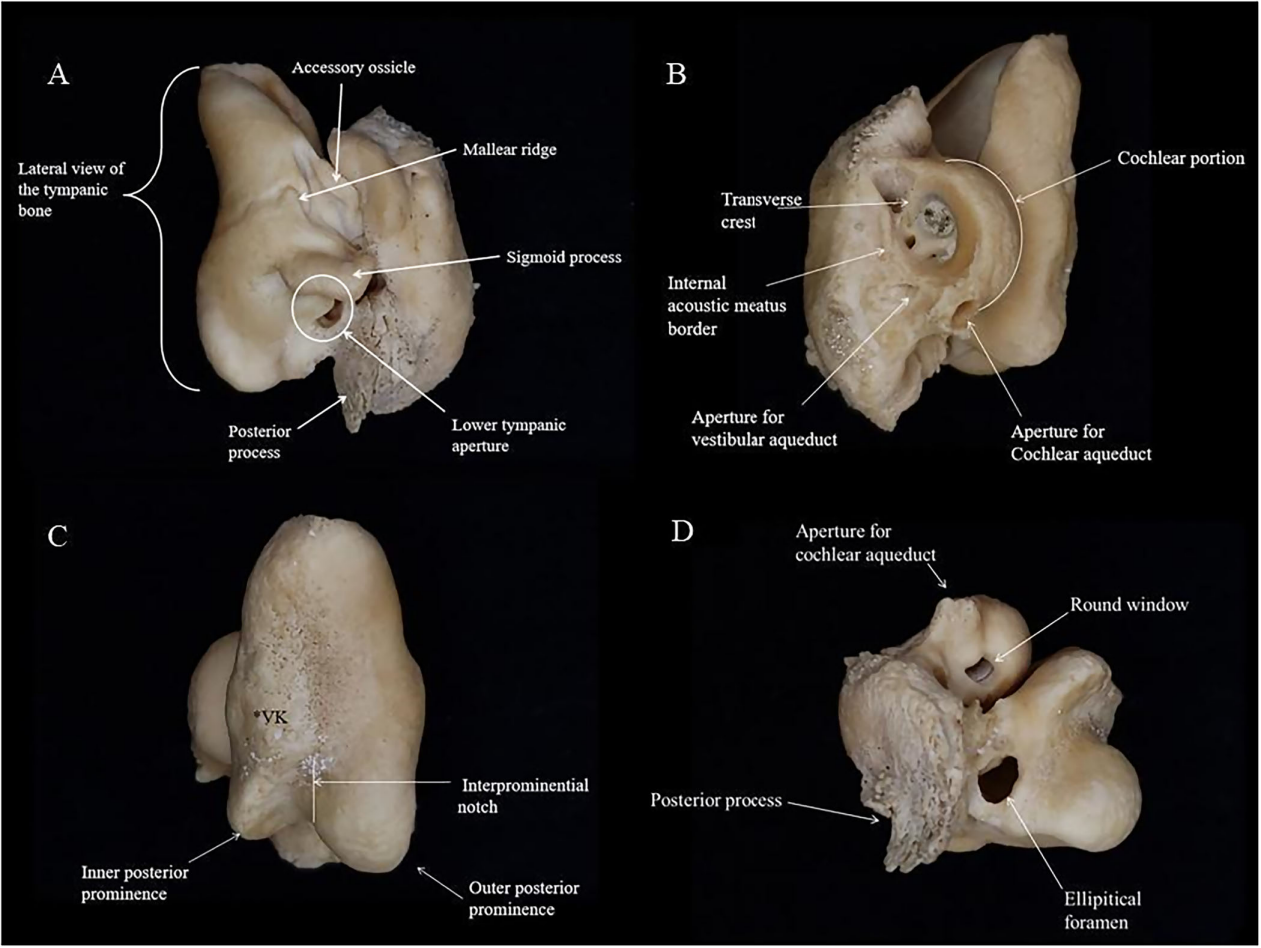
Tympanic‐periotic complex of the species *Sotalia guianensis* with main morphological structures identified. Images in lateral (A), dorsomedial (B), ventral (C), and posterior (D) views, respectively. *VK: Ventral keel.

To describe the morphological patterns found in the recorded species and evaluate potential taxonomic and population variations, the main morphological structures of the complexes were measured and described (Figure 2). The orientation and nomenclature used for the morphological descriptions in this study followed the definitions established by Mead and Fordyce (2009).

For *Sotalia guianensis*, which is represented by a higher number of individuals, the absence of sexual dimorphism in their TPC was confirmed by previous studies (Borobia 1989; Monteiro‐Filho et al. 2002; Arcoverde et al. 2014); the left TPCs were considered because no significant asymmetry between the right and left sides was found (Kasuya 1973; Parente et al. 1999; Arcoverde et al. 2014). Descriptive statistical analyses, including mean, minimum, maximum, standard deviation, and coefficient of variation, were then performed to describe these complexes. To examine a potential relationship between ontogeny and bulla size and to assess the effect of animal length on TPC dimensions, a linear regression analysis was performed, with statistical significance set at *p* < 0.05.

For the other species, due to the small sample size, no statistical analyses were conducted. Measures obtained were used for comparison, evaluating morphological differences among them.

## 3 Results

The smallest measurements obtained for the length of the tympanic bone (M1) and the length of the periotic bone (M14) were from the species *S. longirostris*, with M1 measuring 25.93 mm and M14 measuring 26.31 mm. In contrast, the largest measurements obtained were from the species *P. crassidens*, with M1 measuring 50.35 mm and M14 measuring 44.62 mm. Regarding the width of the TPC (at the maximum point), represented by measurement M24, the same pattern as the length was observed. The largest width was obtained for the species *P. crassidens* with 43.48 mm, and the smallest was obtained for the species *S. longirostris* with 21.48 mm.

The 24 measurements for each TPC obtained from individuals of the species *P. electra, P. crassidens, S. attenuata, S. longirostris*, and *T. truncatus* are given in Table 1.

**Table 1:** Measurements of TPC for the species: *Pseudorca crassidens, Peponocephala electra, Tursiops truncatus, Sotalia guianensis, Stenella attenuata, Stenella longirostris and Sotalia guianensis*.

*Variables*	*Pseudorca crassidens (n* = *2)*		*Peponocephala electra (n* = *4)*				*Tursiops truncatus (n* = *4)*				*Stenella attenuata (n* = *4)*				*Stenella longirostris (n* = *4)*			
	*PSE01*	*PSE02*	*PEP01*		*PEP02*		*TUR01*		*TUR02*		*ATT01*		*ATT02*		*LON01*	*LON02*	*LON03*	
	Left	Right	Left	Right	Left	Right	Left	Right	Left	Right	Left	Right	Left	Right	Right	Left	Left	Right
*Sex*	F	M	NA		F		NA		F		NA		F		F	M	F	
TL	394	489	200		260		NA		99		98		92		84.3	190	179	
M1	48.7	50.35	36.56	35.96	36.93	36.48	36.6	36.95	33.32	33.36	28.52	28.46	28.53	28.41	25.93	28.87	29.44	29.9
M2	43.73	46.24	32.1	32.41	33.69	33.21	35.75	35.75	31.8	31.81	26.86	26.24	27.32	26.84	24.99	28.2	27.84	27.74
M3	36.05	35.15	25.3	26.3	26.48	26.25	24.87	24.97	22.92	22.42	20.55	20.44	20.35	20.22	18.9	20.12	21.16	20.31
M4	27.53	27.21	19.55	20.46	20.52	20.71	19.24	19.46	16.49	16.86	14.57	14.39	15.36	15.25	13.6	15.04	16.6	17.04
M5	30.91	30.27	19.62	20.52	21.36	21.41	20.93	21.49	19.63	20.28	17.42	17.25	17.4	15.35	14.35	17.51	16.44	16.86
M6	38.05	34.73	26.74	27.12	26.05	25.8	25.14	25.64	23.58	23.62	20.69	21.12	21.4	21.14	18.6	21.15	19.72	17.22
M7	25.99	24.61	18.55	18.21	19.89	19.58	18.23	18.23	18.22	17.9	14.54	14.48	14.57	14.47	12.55	13.56	14.35	14.75
M8	10.81	12.79	7.76	8.03	7.77	7.71	7.68	8.24	7.24	7.84	7.69	7.55	7.94	8.25	5.24	7.52	6.28	7.28
M9	5.63	5.64	5.26	5.1	5.05	5.02	5.26	5.5	5.4	5	4.96	4.88	5.31	5.49	4.4	4.51	NA	4.45
M10	15.14	13.21	7.82	8.89	9.45	9.35	9.11	8.31	9.13	9.2	7.87	7.79	7.8	NA	8.5	8.28	NA	8.42
M11	2.4	1.62	1.29	1.32	1.85	1.63	1.65	2.3	2.53	1.48	1.01	1.16	1.16	NA	1.53	1.35	1.6	1.54
M12	8.02	4.28	4.32	4.62	5.02	8.16	5.16	4.43	1.9	3.7	3.21	NA	2.44	NA	2.08	3.44	3.95	2.61
M13	6.21	6.73	3	3.19	5.35	5.23	4.82	5.33	3.63	4.35	3.85	4.01	4.27	NA	3.08	4.5	3.69	3.66
M14	44.62	43.3	34.06	33.85	31.7	32.07	31.47	31.6	31.04	31.54	28.9	28.81	28.02	NA	26.31	26.61	27.08	27.01
M15	15.66	14.12	14.61	14.59	13.6	14.57	12.93	13.15	13.88	12.26	13.13	13.37	10.5	NA	12.51	12.98	12.42	13.4
M16	28.1	28.18	17.8	16.4	18.59	18.79	19.62	22.37	18.33	17.58	14.32	14.16	13.93	NA	14.62	16.88	17.05	15.64
M17	30.65	31.22	23.12	22.66	20.89	21.05	21.48	20.92	21.3	21.59	19.55	20.12	19.51	NA	17.65	18.39	19.1	17.6
M18	3.19	2.19	1.22	1.26	1.13	1.59	2.56	2.32	1.39	1.82	1.57	1.6	1.51	NA	2.7	3.58	3.01	2.56
M19	3.35	3.08	2.66	2.46	1.89	1.76	4.38	4.83	4.7	4.3	3.5	2.98	2.63	NA	3.6	2.16	2.61	2.92
M20	28.62	25.7	18.63	17.24	17.06	16.06	18.51	16.63	13.02	13.46	15.49	15.32	11.88	NA	10.79	12.96	12.01	11.97
M21	20.08	20.97	15.11	16.91	14.65	14.44	13.85	14.13	14.66	15.39	13.75	14.11	14.42	NA	12.46	12.32	11.61	12.1
M22	13.73	12.05	10.45	10.38	11.33	9.58	9.38	9.62	9.51	9.54	7.93	8.32	9.62	NA	8.68	10.3	9.24	8.78
M23	18.44	16.83	15.29	14.25	13.49	13.58	10.94	13.42	11.05	11.84	11.68	11.47	9.08	NA	7.93	11.68	10.24	10.12
M24	43.48	43.42	31.6	31.84	30.93	31.21	29.89	30.05	29.4	29.4	24.94	25.04	25.08	NA	21.48	23.14	22.15	22.56

*Note:* Total length (TL) is in centimeters, and measurements from M1 to M24 are in millimeters. F, female; NA, M; male; Not available.

For *S. guianensis* (*n* = 39), the largest data set available in this study, a descriptive statistical analysis was performed (Table 2). The mean length of the tympanic bone (M1) was 33.88 mm, and the mean length of the periotic bone (M14) was 30.79 mm. The mean width of the tympano‐periotic complex (M24) was 28.41 mm. Raw data related to measurements for *S. guianensis* are provided in the supplementary material (Table S1).

**Table 2:** Descriptive statistics of the TPC from 20 individuals of the species *Sotalia guianensis*, including the mean, standard deviation (SD), minimum (Min), maximum (Max) and coefficient of variation (CV).

Variable	Mean	SD	Mín	Max	CV
M1	33.88	0.91	31.78	35.37	0.026
M2	31.57	0.68	30.23	32.68	0.021
M3	22.58	0.5	21.64	23.45	0.022
M4	16.97	0.48	16.12	17.74	0.028
M5	19.39	0.74	18	20.81	0.038
M6	23.3	0.7	22.12	24.54	0.029
M7	17.56	0.86	16.13	19.02	0.049
M8	8.92	0.89	7.54	11.51	0.099
M9	4.72	0.34	4.15	5.48	0.073
M10	8.63	0.29	8.28	9.18	0.033
M11	1.71	0.22	1.42	2.15	0.127
M12	3.44	0.69	1.81	4.75	0.199
M13	5.24	0.45	4.41	6.18	0.086
M14	30.79	1.3	28.64	33.03	0.042
M15	12.1	0.86	10.77	13.65	0.070
M16	18.88	1.82	15.42	22.03	0.096
M17	19.82	0.56	18.76	20.9	0.028
M18	1.9	0.25	1.5	2.6	0.130
M19	1.93	0.3	1.44	2.69	0.157
M20	14.19	0.83	12.35	15.91	0.058
M21	13.89	0.47	12.82	14.82	0.034
M22	10.75	0.64	9.9	11.77	0.059
M23	13.6	1.37	11.62	17.04	0.100
M24	28.41	0.65	27.22	29.47	0.023

*Note:* The measurements M1‐M24 is shown in millimeters.

The assessment of the effect of the animal's length on the length of the TPC yielded a nonsignificant result (*p* = 0.663), indicating that the variation in the complex's length is not associated with the animal's length in the *S. guianensis* species. The length of the tympanic and periotic bones was similar for juveniles and adult individuals.

### 3.1 Morphological Descriptions of the Tympanic and Periotic Bones

**Figure 3:**
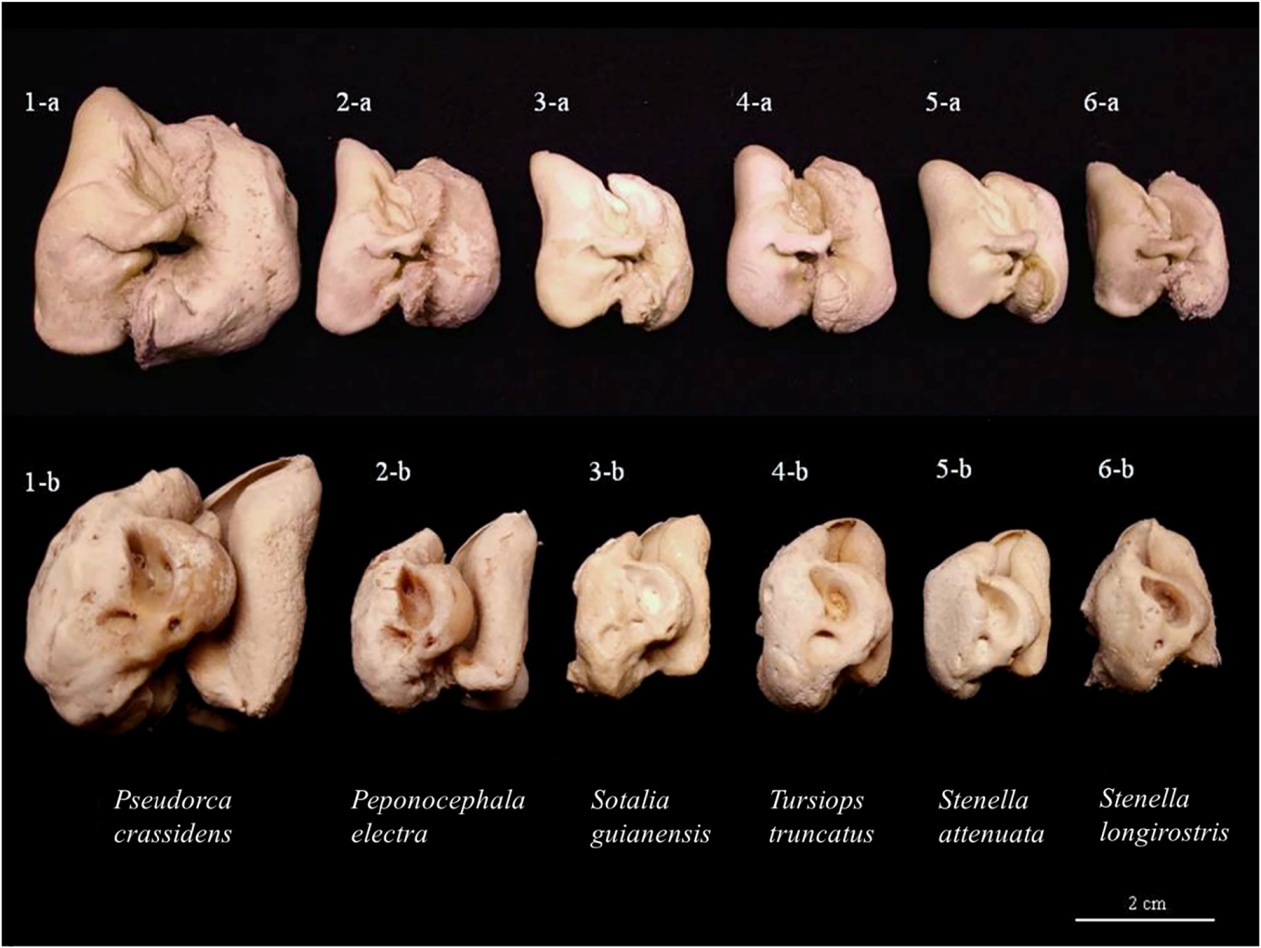
Tympano‐periotic complexes of the 6 recorded species: 1‐ *Pseudorca crassidens* (PSE01), 2‐ *Peponocephala electra* (PEP02), 3‐ *Sotalia guianensis* (SOT13), 4‐ *Tursiops Truncatus* (TUR02), 5‐ *Stenella attenuata* (ATT02), and 6‐ *Stenella longirostris* (LON02) in lateral (a) and dorsomedial (b) views.

To provide an overview of the general morphology of the tympanic and periotic bones in the species analyzed, the tympano‐periotic complexes of all six odontocete species included in this study are presented in Figure 3. The figure illustrates both lateral (a) and dorsomedial (b) views, serving as a comparative reference for the detailed descriptions that follow.

#### 3.1.1 Pseudorca crassidens

The tympanic bone, in lateral view, appears slightly concave (Figure 3–1a). The posterior process is thick and has a base larger than the extremity, resembling a triangle and projecting posterior‐laterally. Its elliptical foramen is open. The lower tympanic aperture is narrow, and its sigmoid process is wide and curved anteriorly. It features a well‐defined malleal crest and a prominent accessory ossicle. Its inner posterior prominence is moderately thin, pointed, and lateralized in the dorsomedial direction, while the outer posterior prominence is thick, prominent, and rounded. The two posterior prominences are separated by a deep interprominential notch.

On the periotic bone, in dorsomedial view (Figure 3–1b), the cochlear portion is prominent, which in this species is globular, thick, and has its apex (the most extreme and thick region) in the central area, similar to the species *T. truncatus* and *P. electra*. Its internal acoustic meatus is deep, with an absent transverse crest and a slightly protruding edge. The aperture of the cochlear aqueduct is characterized by a hole in the posterior region of the cochlear portion and has a slightly prominent edge. Its vestibular aqueduct is characterized by a rounded, wide opening with a rugose appearance. In this species, the round window is located a slightly further, in the anterior direction relative to the cochlear aqueduct, like in *T. truncatus and P. electra*.

#### 3.1.2 Peponocephala electra

The tympanic bone, in lateral view, exhibits a deep concavity (Figure 3–2a). Its posterior process is slender, with length greater than width, resembling a rectangle and projecting laterally. The elliptical foramen is open, and the lower tympanic aperture is broad, with the sigmoid process being slender and curved anteriorly. It features a well‐defined mallear ridge and a prominent accessory ossicle. The inner posterior prominence is moderately thin, with a rounded edge and lateralized in the dorsomedial direction, while the outer posterior prominence is thick and prominent, with a rounded shape directed ventrally. The two posterior prominences are separated by a deep interprominential notch.

As for the periotic bone, in dorsomedial view (Figure 3–2b), it is possible to visualize the cochlear portion, which in this species is globular, thick, and has its apex (most extreme and thick region) in the central area, similar to that in *T. truncatus* and *P. crassidens*. The internal acoustic meatus is deep, with a short or absent transverse crest and a straight edge. The aperture for cochlear aqueduct is characterized by a hole present in the posterior region of the cochlear portion and has a slightly prominent edge, while its vestibular aqueduct is characterized by an irregular, wide, and rugose‐looking opening. In this species, the round window is located slightly further from the cochlear aqueduct, as observed in *T. truncatus* and *P. crassidens*.

When connected, the periotic and tympanic bones of this species appear to be relatively more separated than in the other species described in this study. Additionally, it can be observed that its cochlear portion, in dorsomedial view, is positioned beside the tympanic enclosure, whereas in other species, these structures are generally overlapped.

#### 3.1.3 Sotalia guianensis

The tympanic bone, in lateral view, exhibits a deep concavity (Figure 3–3a). Its posterior process is thick, with a length greater than its width, resembling a rectangle, projecting postero‐laterally, and featuring an undulated contact surface with the tympanic process. It presents an open elliptical foramen and a wide tympanic aperture, while its sigmoid process is thin and straight, showing a slight curvature at its end closest to the periotic bone. It has a well‐defined mallear crest and a prominent accessory ossicle. The inner posterior prominence is moderately thin, rounded, and laterally oriented in the dorsomedial direction, while the outer posterior prominence is thick and prominent, oval shaped. The two posterior prominences are separated by a wide and deep interprominential notch. It features a well‐defined ventral keel with a rugose surface.

In the periotic bone, the globular, thick cochlear portion is observed with the apex (the most extreme and thick region) slightly pointing towards the posterior region (Figure 3–3b), comparable to that in *S. attenuata* and *S. longirostris*. Its internal acoustic meatus is deep, featuring a pointed protuberance near the transverse crest on the border. It has a well‐defined transverse crest. The cochlear aqueduct aperture is characterized by a hole in the posterior region of the cochlear portion and has a prominent border. Its vestibular aqueduct has an irregular, narrow orifice with a smooth surface. In this species, the cochlear window is located close to the cochlear aqueduct, similar to *S. attenuata* and *S. longirostris*.

#### 3.1.4 Tursiops truncatus

**Figure 4:**
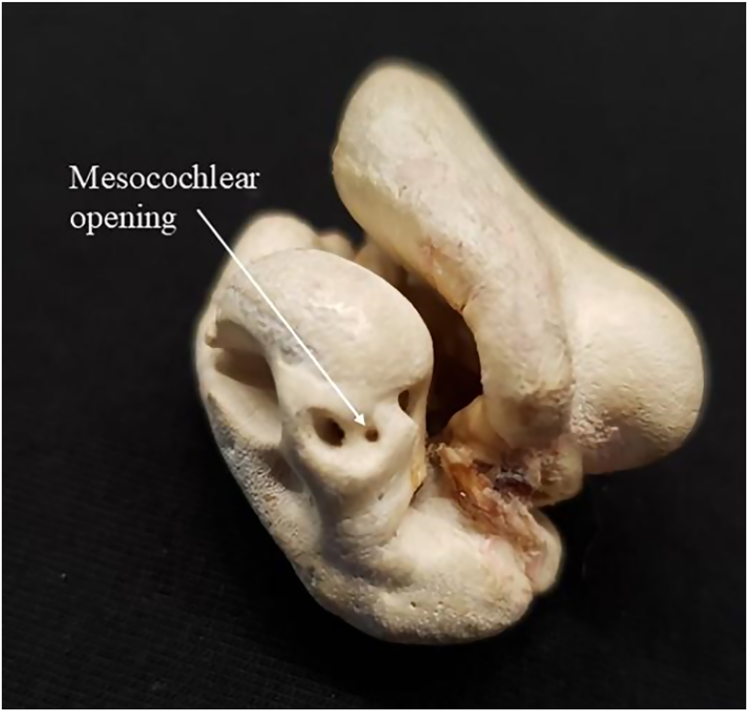
Tympano‐periotic complex of the *Stenella attenuata* species, indicating the structure identified and referred to in this study as the “mesocochlear opening.”

The tympanic bone presents a moderate concavity in lateral view (Figure 3–4a). The posterior process is thick, rounded, and projected laterally and ventrally. Its elliptical foramen is open, the lower tympanic opening is wide, and its sigmoid process is thin and straight, with a slight curvature only at its end close to the periotic bone. It features a well‐defined mallear ridge and prominent accessory ossicle. The inner posterior prominence is moderately thick and rounded, while the outer posterior prominence is thick and prominent, oval shaped. The two posterior prominences are separated by a shallow interprominential notch. It has a slightly marked and slightly rugose ventral keel.

The periotic bone, in dorsomedial view, presents a globular, thick cochlear portion with the apex (most extreme and thick region) in the center (Figure 3–4b), similar to that in *P. electra* and *P. crassidens*. Additionally, its cochlear portion is well‐elevated, and when the tympanic and periotic bones are connected, its position overlaps with the tympanic, unlike in other species where the cochlear portion has a lateral position to the tympanic involucrum. The internal acoustic meatus is deep, featuring a slight protuberance on the edge. It has a present and short transverse crest. The aperture of the cochlear aqueduct is characterized by a hole in the posterior region of the cochlear portion with a prominent edge. Its vestibular aqueduct aperture is a wide, rounded orifice with a smooth surface. In this species, the round window is located far from the aperture of the cochlear aqueduct, similar to that in *P. electra* and *P*. crassidens. The main morphological characteristics of the tympanic and periotic bones of the species recorded in this study are summarized in a table available in the supplementary material (Table S2).

#### 3.1.5 Stenella attenuata

The tympanic bone, in lateral view, exhibits a moderate concavity (Figure 3–5a). Its posterior process is thick, slightly rounded, and projected ventrally, with a serrated surface in contact with the tympanic process. The elliptical foramen is open, and the lower tympanic aperture is wide, while the sigmoid process is thin and curved anteriorly. It features a well‐defined malleus crest and a prominent accessory ossicle. The inner posterior prominence is thin, pointed, and laterally oriented in the dorsomedial direction, while the outer posterior prominence is moderately thick and salient, oval shaped. The two posterior prominences are separated by a superficial interprominential notch. It presents a well‐defined ventral keel with a slightly rugose surface.

Its periotic bone displays a cochlear portion with a globular shape, thickness, and the apex (thickest region) slightly pointed towards the posterior region (Figure 3–5b), similar to *S. guianensis* and *S. longirostris*. The internal acoustic meatus is deep, with a slight protrusion on the edge. It lacks a transverse crest. The aperture for cochlear aqueduct is characterized by a hole located in the posterior region of the cochlear portion, in the posteromedial aspect, with a prominent border all around. The vestibular aqueduct has a narrow, rounded aperture with a smooth surface, and the round window is located close to the aperture for the cochlear aqueduct, similar to *S. guianensis* and *S. longirostris*.

For this species, a small opening was observed positioned between the cochlear aqueduct and the round window, a structure not previously described in earlier studies. Therefore, in this study, it was named “mesocochlear opening” due to its morphological position (Figure 4).

#### 3.1.6 Stenella longirostris

Ossicle in lateral view presents a slight concavity, and its posterior process is thick and projected postero‐laterally, having a base larger than the end, similar to a triangle. Its elliptical foramen is open (Figure 3–6a), and the lower tympanic opening is narrow, with its sigmoid process being thin and curved posteriorly. It shows a marked mallear ridge and a prominent accessory ossicle. Its inner posterior prominence is thin, pointed, and lateralized in the dorsomedial direction, while the outer posterior prominence is moderately thin, similar in size to the internal, pointed in shape, and lateralized towards the posterior process. The two posterior prominences are separated by a deep interprominential notch. It presents a demarcated ventral keel with a slightly rough surface.

In the periotic bone, in dorsomedial view (Figure 3–6b), it is possible to observe that the cochlear portion is well‐elevated and presents a globular, thick shape with the apex (most extreme and thick region) pointing towards the posterior region, as observed in *S. guianensis* and *S. attenuata*. The internal acoustic meatus is deep, showing a slight protrusion at the edge. It has a present and well‐defined transversal crest, and the aperture for cochlear aqueduct is characterized by a hole located in the posterior region of the cochlear portion, presenting a slightly prominent edge. Its vestibular aqueduct has a narrow, rounded aperture with a smooth surface. In this species, the round window is located near the aperture of the cochlear aqueduct, similar to that in *S. guianensis* and *S. attenuata*.

## 4 Discussion

Morphological studies of cetacean TPC have been conducted with the aim of better understanding these structures and their evolutionary (Gutstein et al. 2014), interspecific (Kasuya 1973; Parente et al. 1999; Morell et al. 2007; Moreno 2008), and population‐level variations (Arcoverde et al. 2014; Rigon 2015).

Considering that size, composition, and shape determine the vibrational parameters of any structure (Cranford et al. 2010, 2008; Cranford and Krysl 2012), morphological descriptions of these structures are of utmost importance for understanding variations in auditory frequencies and, consequently, habitat and behavior among different species and populations.

The linear morphometric averages obtained in this study for the tympanic and periotic bones of *S. guianensis* were consistent with those reported in previous research. Parente et al. (1999) analyzed specimens collected in Ceará, while Arcoverde et al. (2014) examined four different populations from the states of Pará, Maranhão, Ceará, and Rio de Janeiro. Among these, the Ceará population exhibited morphometric averages most closely aligned with the findings of the present study.

In the case of the other species recorded in this study, except for *T. truncatus*, which, due to its widespread distribution, has a larger data set for morphological analysis, there are few available studies that encompass the morphometrics of the TPC, allowing for a comparative analysis with this study.

For *T. truncatus*, the minimum and maximum measurements obtained for the length of the tympanic bone and for the length of the periotic bone were similar to the measurements recorded in the study by Rigon (2015) with specimens from Southern Brazil, Argentina, and Uruguay, similar to the measurements recorded in the study by Ketten (1990), conducted with specimens collected in the western North Atlantic, and with the measurements from the study by Parente et al. (1999), conducted with specimens from Ceará. Thus, unlike what was found in *S. guianensis*, it was not possible to observe a similarity in the geographic variation of measurements for *T. truncatus*, obtaining a high variability of minimum and maximum lengths for the complexes in each locality in each study. This condition could be related to the existence of different populations with coastal and/or oceanic preferences.

In the study conducted by Natoli et al. (2004), it was observed that coastal populations of *T. truncatus* showed lower genetic variability and, in most cases, were significantly different from pelagic groups. Additionally, Hohl et al. (2020), assessing cranial variations, found a clear distinction between individuals inhabiting the Atlantic and Pacific Oceans, explaining the high variability between the minimum and maximum measurements obtained in this study.

For *P. electra*, the minimum and maximum measurements obtained in this study were similar to two previous studies (Kasuya 1973; Parente et al. 1999). Kasuya's study (1973) included measurements for specimens collected in Japan, while Parente's study (1999) focused on specimens from Brazil, specifically in Ceará. The similarity in measurements from geographically distant populations may be related to the oceanic and migratory habits of the species, as well as its highly social behavior, living in large aggregations formed by smaller groups. Genetic studies for this species across different populations in the Pacific, Indian, and Atlantic Oceans suggest a relatively high level of interbreeding between populations, indicating constant movement among individuals from different groups and generating a high gene flow (Emery et al. 2017).

For *S. attenuata*, there are currently no available records in the literature that present morphometric data for populations recorded in Brazil. Previous studies have been documented for specimens from Japan (Kasuya 1973), the Eastern Pacific (Perrin 1975), and the Western North Atlantic (Ketten 1990). The minimum and maximum measurements obtained in this study fall within the range of minimum and maximum lengths found in Ketten's study (1990), showing differences from the populations recorded by Kasuya (1973) and Perrin (1975). This variation in measurements for the species may be related to its broad distribution and different habitats, as it can inhabit coastal or oceanic environments.

In Perrin's study (2001), different lengths and cranial measurements were identified for different populations of *S. attenuata*. Coastal subspecies from the Eastern tropical Pacific recorded the largest sizes, while those from the Western Pacific were the smallest.

For *S. longirostris*, morphometric data from populations recorded in Brazil were not found in the literature. The only measurements recorded were in Kasuya's study (1973) with specimens from the Eastern Pacific. The measurements obtained in this study for the length of the tympanic bone and the length of the periotic bone showed a small variation compared to the measurements obtained in Kasuya's study (1973). This variation in measurements is expected and is in line with previous studies that affirm the existence of morphological variations among subspecies. According to Perrin (1990), four subspecies of *S. longirostris* are currently recognized, and the pantropical spinner dolphin is the likely species recorded in this study due to its occurrence in all tropical and subtropical waters worldwide (Jefferson et al. 2011).

Similarly, for *P. crassidens*, no previous studies were found that provided morphometric data for the TPCs of specimens recorded in Brazil. The only measurements recorded were described for populations in the North Atlantic and Pacific in Kasuya's study (1973). In our study, the minimum and maximum measurements obtained for the length of the tympanic and the length of the periotic were within the minimum and maximum standards obtained in Kasuya's study (1973). This similarity between measurements obtained from different populations may be related to their behavior. Like *P. electra, P. crassidens* exhibits social and gregarious behavior, generally seen in large groups with hundreds of individuals (Baird 2009), generating gene flow between different populations.

The six species described in this study are classified as part of the Delphinidae family (LeDuc 2009), and despite many uncertainties about the evolutionary relationships between their genera and species, they share common morphological characteristics, as seen in previous studies by Kasuya (1973), Moreno (2008), Mead and Fordyce (2009), and Wickert (2018).

It was observed that the cochlear portion of the six recorded species has a globular and thick shape, characteristic of species with marine and/or estuarine habitats. According to the study by Gutstein et al. (2014), the cochlear duct is located internally to the cochlear portion, with its base on the ventral surface of the periotic and its apex on the dorsal surface, with these structures being strongly related. Thus, larger sizes of the cochlear portion correspond to larger sizes of the cochlear duct, directly related to a wide range of high and low‐frequency sounds (Gutstein et al. 2014). In the study by Southall et al. (2008), cetacean species were grouped based on their auditory frequency spectrum, and the six species recorded in this study fall into the medium‐frequency group (150 to 160 kHz).

The six species also exhibited thicker outer posterior prominences than internal posterior prominences, consistent with what was previously reported by Kasuya (1973) for the Delphinidae. However, despite displaying this pattern, the inner and outer posterior prominences showed different proportions and shapes for different species. Additionally, as in the studies by Kasuya (1973) and Parente et al. (1999), the species described here had posterior processes projected laterally, postero‐laterally, or ventrally, which may be an important morphological characteristic to assist in species and/or population identification.

Some small variations were also observed in the positions of the apices (thicker portion) of the cochlear region, with the species *S. guianensis, S. attenuata*, and *S. longirostris* having apices directed towards the posterior region of the periotic, while the *species P. electra, P. crassidens*, and *T. truncatus* had a central apex. Another characteristic observed in all species recorded in this study, except for *P. electra*, is the prominent edge of the internal acoustic meatus, which had been previously reported by Moreno (2008) for the species *T. truncatus, S. attenuata*, and *S. longirostris*, as a feature of the Delphinidae. In the cochlear aqueduct, a prominent edge was observed in all species, except for *P. electra* and *P. crassidens*.

Therefore, regarding the structures of the periotic bones, the species *S. guianensis*, *S. attenuata*, and *S. longirostris* showed a greater number of similar characteristics, including: the apex of the cochlear region in the central position, the narrow width of the vestibular aqueduct, the smooth surface of the transverse crest, and the closer proximity between the aperture of the cochlear aqueduct and the round window.

These similarities may reflect taxonomic or functional proximity, potentially associated with shared ecological traits, such as habitat use and acoustic behavior, among these species. A highlight in this study was the observation of a structure in the periotic bone of *S. attenuata*—an opening between the cochlear aqueduct and the cochlear window, not yet described in the literature. This structure was named in this study as the “mesocochlear opening” (Figure 4). The function of this opening remains uncertain. This opening may be a morphological variation in this specific population, and further studies are needed for a definitive characterization combining several imaging techniques.

In summary, the morphological characteristics considered most representative for species determination, based on the macroscopical observation of the bone structures of the TPCs, would be, 1) for the tympanic bone: the posterior process, the inner and outer prominences, and the sigmoid process; and 2) for the periotic bone: the cochlear portion, the aperture of the cochlear and vestibular aqueducts, and the transverse crest. These characteristics proved to be of great importance because, despite showing morphological variations among different species, they exhibited little individual variation, which could be a key point in identifying taxonomic and population patterns.

Although the structures analyzed in this study are not directly involved in frequency discrimination mechanisms, the tympanic and periotic bones are essential components of the sound transmission pathway in odontocetes. Their morphology influences how vibrations are propagated to the inner ear, and their density and geometry may affect acoustic impedance and mechanical conduction (Cranford et al. 2010).

In addition to the functional context, our results regarding the absence of ontogenetic variations in the morphometric analysis of the tympanic and periotic bones found in this study for *S. guianensis*, as well as the similarity observed between the measurements obtained for the tympanic and periotic bones of juvenile and adult individuals of *P. electra* and *S. longirostris* species, were consistent with previous studies. de Buffrénil et al. (2004) demonstrated that at birth, the growth of the tympanic and periotic bones is already complete, suggesting early ossification and minimal postnatal morphological change in these structures. Similarly, Perrin (1975), in a comprehensive morphometric study of *Stenella* species with a large sample size from the eastern tropical Pacific and Hawaii, also reported limited ontogenetic variation in cranial and earbone measurements. These findings support the hypothesis that, in delphinids, the tympano‐periotic complex reaches its definitive form early in development, making it a reliable structure for comparative morphological and taxonomic studies.

## 5 Conclusion

The description of the TPC of *Peponocephala electra, Pseudorca crassidens, Sotalia guianensis, Stenella attenuata, Stenella longirostris*, and *Tursiops truncatus*, from Rio Grande do Norte, allows the following conclusions:

- The six studied species shared similar morphological characteristics, with a notable emphasis on *S. guianensis, S. attenuata*, and *S. longirostris*, suggesting a higher degree of morphological similarity among them.
- The most representative morphological characteristics for species identification were, for the tympanic bone: the posterior process, inner and outer prominences, and the sigmoid process. For the periotic bone, they were: the cochlear portion, aperture of the cochlear and vestibular aqueducts, and the transverse crest.
- Macroscopic analysis identified an opening between the cochlear aqueduct and the cochlear window, in individuals of *S. attenuata* species, not previously described in the literature. This structure is named “mesocochlear opening” in this study.
- No apparent ontogenetic variations were observed in the TPC for *S. guianensis*, *P. electra*, and *S. longirostris*, which provided data for different age groups.

## Author Contributions

**Gabriela Colombini‐Corrêa:** investigation, writing – original draft, methodology, data curation, formal analysis, conceptualization. **Maria Morell:** writing – review and editing, supervision. **Ana Bernadete Lima Fragoso:** writing – review and editing, resources, supervision. **Daniel Solon Dias de Farias:** writing – review and editing, supervision. **Flávio José de Lima Silva:** data curation, resources, writing – review and editing. **Simone Almeida Gavilan:** supervision, resources, project administration, data curation, writing – review and editing.

## 1 Peer Review

The peer review history for this article is available at https://www.webofscience.com/api/gateway/wos/peer-review/10.1002/jmor.70089.

## Supporting information


**Table S1:** Measurements of the TPC of the species *Sotalia guianensis*. The total Length (TL) is shown in centimeters. The measurements from M1 to M24 are shown in millimeters. *NA= Not Available.

**Tabel S2:** Morphological characterization of the tympanic and periotic bones of the six species described in this study.

## Data Availability

The data that support the findings of this study are available on request from the corresponding author. The data are not publicly available due to privacy or ethical restrictions.
