# Supplementary material for: Morphology of the Tympano‐Periotic Complex in Stranded Odontocetes in Northeast Brazil
Source: J Morphol. 2025 Sep 26;286(10):e70089. doi: 10.1002/jmor.70089 (PMC12464893; doi:10.1002/jmor.70089)
Supplement: Supplementary file 1 — Table S1: Measurements of the TPC of the species Sotalia guianensis. The total Length (TL) is shown in centimeters. The measurements from M1 to M24 are shown in millimeters. *NA= Not Available. [file JMOR-286-e70089-s002.docx]

**SUPPLENTARY MATERIAL**

**Table S1** "Measurements of the TPC of the species *Sotalia guianensis*. The total Length (TL) is shown in centimeters. The measurements from M1 to M24 are shown in millimeters. *NA= Not Available."

| Variable | ***Sotalia guianensis (N=39)*** | | | | | | | | | | |
| --- | --- | --- | --- | --- | --- | --- | --- | --- | --- | --- | --- |
|  | *SOT01* | | *SOT02* | | *SOT03* | *SOT04* | | *SOT05* | | *SOT06* | |
|  | left | right | left | right | left | left | right | left | right | left | right |
| Sex | FEM | FEM | FEM | FEM | MAL | IND | IND | IND | IND | FEM | FEM |
| *TL* | 128.00 | 128.00 | 170.40 | 170.40 | 194.00 | 178.00 | 178.00 | 150.00 | 150.00 | 185.00 | 185.00 |
| M1 | 33.49 | 33.78 | 33.12 | 32.63 | 33.80 | 34.07 | 34.12 | 34.57 | 34.37 | 34.56 | 34.95 |
| M2 | 31.91 | 32.14 | 31.29 | 31.05 | 32.80 | 31.70 | 31.86 | 31.22 | 31.03 | 31.71 | 31.91 |
| M3 | 23.00 | 22.83 | 22.47 | NA | 22.16 | 22.35 | 22.45 | 22.40 | 22.51 | NA | 23.41 |
| M4 | 17.36 | 17.83 | 17.10 | NA | 17.07 | 17.07 | 17.18 | 16.25 | 17.00 | 17.45 | 17.44 |
| M5 | 19.49 | 19.76 | 19.56 | 20.40 | 20.31 | 19.95 | 19.85 | 18.86 | 18.68 | 19.66 | 19.23 |
| M6 | 23.81 | 24.24 | 23.22 | NA | 19.60 | 24.26 | 24.22 | 22.68 | 22.45 | NA | 24.54 |
| M7 | 18.03 | 18.41 | 17.43 | 17.15 | 16.79 | 17.76 | 19.85 | 16.76 | 16.80 | 19.17 | 18.88 |
| M8 | 8.93 | 8.59 | 9.40 | 7.78 | 9.83 | 12.57 | 10.45 | 7.78 | 8.66 | 7.79 | 7.29 |
| M9 | 4.65 | 4.51 | 4.47 | NA | 3.68 | 4.80 | 5.21 | 4.45 | 4.42 | NA | 4.92 |
| M10 | 9.28 | 8.48 | 8.38 | NA | 9.10 | 8.96 | 9.08 | 8.95 | 8.99 | NA | 8.77 |
| M11 | 1.15 | 1.88 | 1.50 | NA | 1.52 | 1.56 | 1.46 | 2.22 | 2.08 | 1.77 | 1.19 |
| M12 | 4.80 | 4.70 | 3.41 | 3.92 | 2.70 | 3.07 | 3.09 | 3.11 | 3.12 | 2.24 | 1.37 |
| M13 | 5.80 | 5.46 | 6.27 | 6.10 | 4.98 | 5.25 | 5.15 | 5.29 | 4.96 | 5.30 | 5.03 |
| M14 | 31.45 | 30.16 | 31.47 | 31.18 | 29.52 | 28.55 | 28.73 | 29.36 | 29.09 | 34.05 | 31.76 |
| M15 | 12.28 | 12.67 | 13.30 | 12.76 | 11.86 | 13.01 | 10.21 | 12.14 | 10.88 | 13.81 | 13.49 |
| M16 | 20.21 | 23.85 | 15.20 | 19.79 | 13.89 | 14.42 | 19.07 | 17.25 | 16.41 | 18.56 | 17.02 |
| M17 | 20.26 | 20.26 | 19.22 | 19.48 | 19.38 | 19.28 | 18.25 | 20.38 | 19.73 | 20.18 | 20.32 |
| M18 | 1.63 | 1.65 | 1.62 | 1.92 | 1.76 | 2.19 | 1.72 | 2.11 | 1.99 | 1.92 | 1.61 |
| M19 | 1.48 | 1.81 | 1.30 | 2.02 | 3.06 | 2.07 | 2.09 | 2.33 | 1.62 | 2.16 | 2.35 |
| M20 | 13.28 | 13.90 | 14.77 | 14.08 | 12.77 | 15.88 | 14.00 | 13.84 | 13.50 | 15.05 | 14.77 |
| M21 | 14.34 | 13.37 | 13.06 | 12.59 | 15.45 | 15.32 | 14.32 | 13.66 | 13.17 | 14.37 | 14.30 |
| M22 | 11.65 | 11.58 | 9.07 | 10.91 | 11.19 | 10.50 | 11.58 | 10.23 | 9.56 | 10.99 | 11.39 |
| M23 | 13.48 | 14.11 | NA | NA | NA | NA | NA | 12.79 | 13.50 | 16.70 | 17.37 |
| M24 | 28.68 | 29.44 | NA | NA | NA | NA | NA | 27.90 | 28.40 | 28.53 | 28.14 |

| Variables | ***Sotalia guianensis (N=39)*** | | | | | | | | | | | | | |
| --- | --- | --- | --- | --- | --- | --- | --- | --- | --- | --- | --- | --- | --- | --- |
|  | *SOT07* | | *SOT08* | | *SOT09* | | *SOT10* | | *SOT11* | | *SOT12* | | *SOT13* | |
|  | left | right | left | right | left | right | left | right | Left | right | left | right | left | right |
| *Sex* | F | F | IND | IND | F | F | M | M | M | M | M | M | M | M |
| *TL* | 175.00 | 175.00 | 181.00 | 181.00 | 139.00 | 139.00 | 131.00 | 131.00 | 115.00 | 115.00 | 192.00 | 192.00 | 132.00 | 132.00 |
| M1 | 34.99 | 34.56 | 34.72 | 34.70 | 32.85 | 32.99 | 32.77 | 32.55 | 33.45 | 33.60 | 34.51 | 34.53 | 33.77 | 33.43 |
| M2 | 32.51 | 32.20 | 32.42 | 32.38 | 30.82 | 30.79 | 31.21 | 30.88 | 31.35 | 30.92 | 32.42 | 31.92 | 31.21 | 31.22 |
| M3 | 23.49 | 23.41 | 22.86 | 22.91 | 22.26 | 22.48 | 22.20 | 22.05 | 22.59 | 22.57 | 23.08 | 23.21 | 22.42 | 22.28 |
| M4 | 17.46 | 17.32 | 17.74 | 17.43 | 16.74 | 16.67 | 16.32 | 16.70 | 16.66 | 16.97 | 16.95 | 17.48 | 16.44 | 16.74 |
| M5 | 18.94 | 19.31 | 20.02 | 19.88 | 19.98 | 19.53 | 18.73 | 19.16 | 17.64 | 18.36 | 18.89 | 19.46 | 19.15 | 19.51 |
| M6 | 23.11 | 23.72 | 23.72 | 23.90 | 23.01 | 22.40 | 23.30 | 22.55 | 23.12 | 22.45 | 21.58 | 22.67 | 23.42 | 22.76 |
| M7 | 18.28 | 17.80 | 18.61 | 18.00 | 17.33 | 16.48 | 16.36 | 16.65 | 16.10 | 16.16 | 16.75 | 16.82 | 17.81 | 17.96 |
| M8 | 8.82 | 8.95 | 9.40 | 9.17 | 8.59 | 7.58 | 8.82 | 8.34 | 8.23 | 8.76 | 8.11 | 8.51 | 9.02 | 8.46 |
| M9 | 4.80 | 4.41 | 4.30 | 4.36 | 4.22 | 5.40 | 5.34 | 4.73 | 5.35 | 5.60 | 4.54 | 4.61 | 4.05 | 4.26 |
| M10 | 8.69 | 8.18 | 8.93 | 8.86 | 8.57 | 8.31 | 8.73 | 8.49 | 8.27 | 8.30 | 8.56 | 8.30 | 8.57 | 8.08 |
| M11 | 1.64 | 1.76 | 1.53 | 1.60 | 1.83 | 1.72 | 1.88 | 1.97 | 1.98 | 2.01 | 1.78 | 1.72 | 1.27 | 1.58 |
| M12 | 3.03 | 3.32 | 3.93 | 4.00 | 3.83 | 3.17 | 3.93 | 4.30 | 3.57 | 4.12 | 4.08 | 3.11 | 3.43 | 3.80 |
| M13 | 5.62 | 5.43 | 5.70 | 5.38 | 4.93 | 4.37 | 5.25 | 5.30 | 4.51 | 4.31 | 5.33 | 4.84 | 5.27 | 5.30 |
| M14 | 32.70 | 30.26 | 32.17 | 30.56 | 30.33 | 32.54 | 29.05 | 29.50 | 31.01 | 30.50 | 30.97 | 30.25 | 29.07 | 29.62 |
| M15 | 12.22 | 11.87 | 10.89 | 10.65 | 13.19 | 13.70 | 11.54 | 11.35 | 12.89 | 12.78 | 11.60 | 10.66 | 12.45 | 14.20 |
| M16 | 19.13 | 18.87 | 19.27 | 20.67 | 19.40 | 20.21 | 17.26 | 18.49 | 20.58 | 21.12 | 17.19 | 16.93 | 17.53 | 17.76 |
| M17 | 20.50 | 19.92 | 20.04 | 19.66 | 19.02 | 19.58 | 19.62 | 19.97 | 19.43 | 19.12 | 20.70 | 21.11 | 20.21 | 20.24 |
| M18 | 2.28 | 2.11 | 1.90 | 1.84 | 1.87 | 1.45 | 1.82 | 1.96 | 1.90 | 2.09 | 1.74 | 1.91 | 1.88 | 2.27 |
| M19 | 1.90 | 1.72 | 2.34 | 2.20 | 1.75 | 1.78 | 1.83 | 1.93 | 1.31 | 1.57 | 2.14 | 1.80 | 2.00 | 1.71 |
| M20 | 14.77 | 15.08 | 14.46 | 14.12 | 13.87 | 13.01 | 13.67 | 13.87 | 13.59 | 13.23 | 14.84 | 14.89 | 14.61 | 13.55 |
| M21 | 14.09 | 13.07 | 13.92 | 13.77 | 14.25 | 14.21 | 12.95 | 14.72 | 13.75 | 13.79 | 13.80 | 13.41 | 14.63 | 14.40 |
| M22 | 10.41 | 9.75 | 11.14 | 10.60 | 11.21 | 11.03 | 10.07 | 9.74 | 10.79 | 11.11 | 10.32 | 10.29 | 10.84 | 10.14 |
| M23 | 13.46 | 14.31 | 15.31 | 14.62 | 12.30 | 12.58 | 11.79 | 12.92 | 11.69 | 11.55 | 12.99 | 13.42 | 13.99 | 13.38 |
| M24 | 27.95 | 28.31 | 29.40 | 29.54 | 27.82 | 27.84 | 28.03 | 27.86 | 27.60 | 28.00 | 28.71 | 29.65 | 28.71 | 28.93 |

| Variables | ***Sotalia guianensis (N=39)*** | | | | | | | | | | | | | |
| --- | --- | --- | --- | --- | --- | --- | --- | --- | --- | --- | --- | --- | --- | --- |
|  | *SOT14* | | *SOT15* | | *SOT16* | | *SOT17* | | *SOT18* | | *SOT19* | | *SOT20* | |
|  | left | right | left | right | left | right | left | right | left | right | left | right | left | right |
| *Sex* | M | M | F | F | M | M | M | M | IND | IND | M | M | M | M |
| *TL* | 127.00 | 127.00 | 196.00 | 196.00 | 87.00 | 87.00 | 120.00 | 120.00 | 190.00 | 190.00 | 127.00 | 127.00 | 151.40 | 151.40 |
| M1 | 34.45 | 34.20 | 35.03 | 34.23 | 35.68 | 35.06 | 34.37 | 34.27 | 32.99 | 33.60 | 31.79 | 31.78 | 33.11 | 33.86 |
| M2 | 32.41 | 32.96 | 31.81 | 32.01 | 32.66 | 31.97 | 31.74 | 31.60 | 30.60 | 30.56 | 30.18 | 30.28 | 31.22 | 31.53 |
| M3 | 22.88 | 21.80 | 22.83 | 23.35 | NA | 23.11 | 22.14 | 22.45 | 21.99 | 21.80 | 21.41 | 21.86 | 22.12 | 22.22 |
| M4 | 17.50 | 16.96 | 17.14 | 17.15 | 17.68 | 17.81 | 16.53 | 16.70 | 16.31 | 15.93 | 16.14 | 16.21 | 16.62 | 16.74 |
| M5 | 20.87 | 20.75 | 19.71 | 19.59 | 20.69 | 20.64 | 18.83 | 19.05 | 18.48 | 17.67 | 18.38 | 19.18 | 19.47 | 19.46 |
| M6 | 24.29 | 23.44 | 23.40 | 23.37 | NA | 24.51 | 22.93 | 23.07 | 23.08 | 21.72 | 22.66 | 23.54 | 22.49 | 23.53 |
| M7 | 18.38 | 18.57 | 18.56 | 18.10 | 18.17 | 18.17 | 17.04 | 17.18 | 16.96 | 16.73 | 16.81 | 16.40 | 17.44 | 17.41 |
| M8 | 9.68 | 10.01 | 9.39 | 9.97 | 8.72 | 8.08 | 10.19 | 10.14 | 8.53 | 8.73 | 9.04 | 8.88 | 8.38 | 9.18 |
| M9 | 4.27 | 4.81 | 4.99 | 5.37 | NA | 5.14 | 4.47 | 4.30 | 4.38 | 4.84 | 4.63 | 5.27 | 4.41 | 4.48 |
| M10 | 8.37 | 8.25 | 8.84 | 9.09 | NA | 8.83 | 9.30 | 9.05 | 8.44 | 8.20 | 8.40 | 8.50 | 8.39 | 8.55 |
| M11 | 1.85 | 1.50 | 2.01 | 2.10 | 1.57 | 1.67 | 1.62 | 1.88 | 1.79 | 1.78 | 1.44 | 1.44 | 1.72 | 2.14 |
| M12 | 3.79 | 2.25 | 3.65 | 4.17 | 1.70 | 3.58 | 3.38 | 3.60 | 4.41 | 3.95 | 3.65 | 3.40 | 0.88 | 3.84 |
| M13 | 6.30 | 6.00 | 5.71 | 4.75 | 5.12 | 5.05 | 4.92 | 5.35 | 4.47 | 4.90 | 4.75 | 4.88 | 5.53 | 5.39 |
| M14 | 33.04 | 31.63 | 31.26 | 32.34 | 30.22 | 32.26 | 32.82 | 33.24 | 32.08 | 29.41 | 28.83 | 29.14 | 29.61 | 29.72 |
| M15 | 11.68 | 12.01 | 11.43 | 12.28 | 13.48 | 11.62 | 12.65 | 11.94 | 10.72 | 11.42 | 11.09 | 11.56 | 10.91 | 12.51 |
| M16 | 15.47 | 15.38 | 21.59 | 20.97 | 21.15 | 19.85 | 20.64 | 21.54 | 19.38 | 18.88 | 18.99 | 20.50 | 18.84 | 17.88 |
| M17 | 19.82 | 19.65 | 19.95 | 20.63 | 19.32 | 20.65 | 19.27 | 19.12 | 19.12 | 18.67 | 20.50 | 20.20 | 20.35 | 19.49 |
| M18 | 2.32 | 2.87 | 1.76 | 1.53 | 1.47 | 1.52 | 1.63 | 1.89 | 1.94 | 2.07 | 2.19 | 2.00 | 1.98 | 1.64 |
| M19 | 1.75 | 2.01 | 1.98 | 1.93 | 1.60 | 1.53 | 1.59 | 1.69 | 2.04 | 2.11 | 2.54 | 2.84 | 2.06 | 2.57 |
| M20 | 12.14 | 12.56 | 14.64 | 14.95 | 16.82 | 15.00 | 15.55 | 14.33 | 15.21 | 13.54 | 13.71 | 13.20 | 13.57 | 13.55 |
| M21 | 14.96 | 13.26 | 13.13 | 14.30 | 14.34 | 14.94 | 13.73 | 13.68 | 14.23 | 13.67 | 13.58 | 13.72 | 13.88 | 13.15 |
| M22 | 11.62 | 11.90 | 10.49 | 11.27 | 10.69 | 12.85 | 10.95 | 10.63 | 11.39 | 11.53 | 10.40 | 9.72 | 10.29 | 10.05 |
| M23 | 14.75 | 13.92 | 15.25 | 15.01 | 14.50 | 14.71 | 11.97 | 12.74 | 14.01 | 14.12 | 12.66 | 11.46 | 11.81 | 13.35 |
| M24 | 28.76 | 28.40 | 29.06 | 28.81 | 29.20 | 29.46 | 27.63 | 27.43 | 27.23 | 27.22 | 28.11 | 28.08 | 28.51 | 28.56 |

*NA = Not available**.** F: female, M: male, IND: indetermined
