# Supplementary material for: Morphology of the Tympano‐Periotic Complex in Stranded Odontocetes in Northeast Brazil
Source: J Morphol. 2025 Sep 26;286(10):e70089. doi: 10.1002/jmor.70089 (PMC12464893; doi:10.1002/jmor.70089)
Supplement: Supplementary file 2 — Tabel S2: Morphological characterization of the tympanic and periotic bones of the six species described in this study. [file JMOR-286-e70089-s001.docx]

**Tabel S2** Morphological characterization of the tympanic and periotic bones of the six species described in this study.

| **Morphological characterization** | | | | | | |
| --- | --- | --- | --- | --- | --- | --- |
| Features | *Peponocephala electra* | *Pseudorca crassidens* | *Sotalia guianensis* | *Stenella attenuata* | *Stenella longirostris* | *Tursiops truncatus* |
| **Tympanic bone** | | | | | | |
| **Lateral view of the tympanic bone** | Deeply concave | Slightly concave | Deeply concave | Moderately concave | Slightly concave | Moderately concave |
| **Posterior process** | Thin, rectangular, and laterally projected | Thick, triangular, and posterior-laterally projected | Thick, rectangular, and posterior-laterally projected. | Thick, rounded, and ventrally projected | Thick, triangular, and posterior-laterally projected | Thick, rounded, and ventrally projected |
| **Inner posterior prominence** | Moderately thin and rounded | Moderately thin and pointed | Thin and rounded | Thin and pointed | Thin and pointed | Moderately thick and rounded |
| **Outer posterior prominence** | Thick, round, and prominent | Thick, round, and prominent | Thick, oval, and prominent | Moderately thick and oval | Moderately thin and pointed | Thick and oval |
| **Interprominential notch** | Deep | Deep | Deep | Superficial | Deep | Superficial |
| **Lower tympanic opening** | Wide | Narrow | Wide | Wide | Narrow | Wide |
| **Mallear ridge** | Marked | Marked | Marked | Marked | Marked | Marked |
| **Accessory ossicle** | Prominent | Prominent | Prominent | Prominent | Prominent | Prominent |
| **Sigmoid process** | Thin and curved anteriorly | Wide and curved anteriorly | Thin and straight | Thin and curved anteriorly | Thin and curved posteriorly | Thin and straight |
| **Elliptical foramen** | Open | Open | Open | Open | Open | Open |
| **Periotic bone** | | | | | | |
| **Cochlear portion** | Globular and thick, with a central apex | Globular and thick, with a central apex | Globular and thick, with the apex pointed towards the posterior region | Globular and thick, with the apex pointed towards the posterior region | Globular and thick, with the apex pointed towards the posterior region | Globular and thick, with a central apex |
| **Aperture for cochlear**  **aqueduct** | Slightly prominent edge | Slightly prominent edge | Prominent edge | Prominent edge (opening between the aqueduct and the cochlear window) | Slightly prominent edge | Prominent edge |
| **Aperture for vestibular aqueduct** | Large, irregular, and rough | Wide, rounded, and rough | Narrow, irregular, and smooth | Narrow, rounded, and smooth | Narrow, rounded, and smooth | Narrow, rounded, and smooth |
| **Transvere crest** | Rough | Rough | Smooth | Smooth | Smooth | Rough |
| **Round window** | Farther from the cochlear aqueduct | Farther from the cochlear aqueduct | Closer to the cochlear aqueduct | Closer to the cochlear aqueduct | Closer to the cochlear aqueduct | Farther from the cochlear aqueduct |
| **Internal acoustic meatus border** | Rectilinear | Slight protrusion | Pointed protrusion at the level of the transverse crest | Slight protrusion | Slight protrusion | Slight protrusion |
| **Transverse crest** | Absent / Short | Absent | Present | Absent | Present | Present |
